# Study of the Effect of Curing Residual Stress on the Bonding Strength of the Single Lap Joint Using a High-Temperature Phosphate Adhesive

**DOI:** 10.3390/ma11071198

**Published:** 2018-07-12

**Authors:** Chengkun Ma, Yuan Tian, Yan Gong, Jifeng Zhang, Hui Qi, Chao Wang

**Affiliations:** 1 Smart Structures and Advanced Composite Materials Lab, College of Aerospace and Civil Engineering, Harbin Engineering University, Harbin 150001, China; mack@hrbeu.edu.cn (C.M.); tian_yuan@hrbeu.edu.cn (Y.T.); qihui@hrbeu.edu.cn (H.Q.); 2 School of Materials Science and Engineering, Beijing Institute of Fashion Technology, Beijing 100029, China; clygy@bift.edu.cn; 3 Institute of Petrochemistry, Heilongjiang Academy of Science, Harbin 150001, China; 13945092540@163.com

**Keywords:** phosphate adhesive, residual stress, bonding strength, micro-Raman spectroscopy, single lap joint, DSC-TG analysis

## Abstract

High-temperature phosphate adhesives are widely used in the aerospace and nuclear power industries. However, complex residual stresses can result when the curing temperature parameters are unreasonable due to the brittleness of the adhesive. To reveal the curing temperature mechanism affecting the bonding strength of the phosphate adhesives, several curing temperature curves (CT-1~6) were designed for the single lap joint (SLJ) using phosphate adhesive. The residual stress helped to reveal the relationship between the curing temperature parameters and the bonding performance. In this process, the residual stress of the silicon carbide joint was measured using micro-Raman spectroscopy, and the tensile strength of the joint was tested. A cohesive zone model (CZM) was established with Abaqus^®^ to verify the results, and the numerical results from the model agreed well with the experimental values. The residual stress and adhesive strength were obviously affected by curing temperature. The reasonable curing temperature curves have the benefits of reducing the residual stress and improving the bonding strength.

## 1. Introduction

High-temperature bonding structures are widely used in the aerospace and nuclear energy industries, in which high-temperature phosphate adhesives are one type of prospective inorganic adhesive. However, unreasonable curing temperature parameters can lead to complex residual stress in the bonding structures [1,2]. Thermal residual stresses result from the mismatch of thermal expansion coefficients between the adhesive and adherents, which affects bonding strength and structural stability [3]. Therefore, regulating the residual stress of the joint is a routine method for obtaining stable bonding performances [4].

Ample research has been performed on the residual stress in bonding structures [5,6,7,8]. Xiao et al. found that residual stress could be reduced by starting the bonding while avoiding the corner of the path, turning the laser off gradually and heating the glass substrates [9]. Seung et al. used electro-spun meta-aramid nanofiber mats to reinforce the epoxy adhesive and reduce the residual stresses caused by mismatch [10]. Djokic et al. discussed the effect of the residual stress produced during the curing process on composite repair [11]. Willemse et al. used high-resolution neutron diffraction and X-ray diffraction to measure the stress in a SiC/Ti 1100 matrix composite material plate [12]. Apalak et al. used incremental FEM for the geometrical nonlinear stress analysis of the single lap joint (SLJ) on four of the adherent edge conditions. Their work concluded that the thermal and mechanical mismatches of the adhesives and adherents caused high strain concentrations through the adhesive regions, which were close to the adhesive–adherent interfaces around the adhesive free ends [13]. Goglio et al. used an instrumented impact pendulum to study the impacts of an epoxy adhesive, and an alternative design approach was given by the evaluation of the stress intensity factor (SIF) [14]. The main advantages of micro-Raman spectroscopy are its easy use and its ability to identify β−SiC and α−SiC crystals [15]. Digregorio et al. used Raman spectroscopy to measure residual stress in the SiC near the surface of the composites and found that the measured decrease in stress with increasing packing fraction is consistent with theoretical predictions based on micromechanics [16], as with several other relevant studies [17,18,19,20,21].

In this paper, several curing temperature curves were designed, and an SLJ was prepared using an inorganic phosphate adhesive with SiC pieces as adherents. Micro-Raman spectroscopy was used to determine the residual stresses in the SiC pieces by measuring the characteristic E_2_-TO peak shift with respect to the typical Raman peak for stress-free SiC at 785 cm^−1^; the shear strength of the joint was also tested. Numerical simulation was used to predict the residual stresses, as well as the bonding strength, of the adhesive. Finally, the relationships between the curing temperature curves, curing residual stress and bonding strength were revealed.

## 2. Experimental

### 2.1. Materials and SLJ Preparation

**Figure 1:**
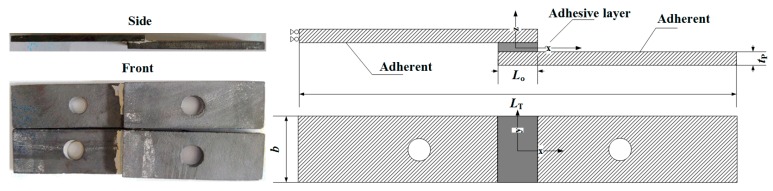
Geometry and characteristic dimensions of the single lap junction (SLJ).

Inorganic phosphate adhesive has the advantages of a low expansion coefficient, corrosion resistance, and high temperature resistance and is widely used in aerospace. The base material used in the adhesive was aluminum dihydrogen phosphate (Al(H_2_PO_4_)_3_). The curing agent is mainly composed of aluminum oxide (Al_2_O_3_), and a corrosion inhibitor, pH regulator, curing promoter, etc., are then added, before mixing and grinding the sample thoroughly. Then, the curing agent is blended with the resin and cured at 180 °C for 1 h [5]. SiC pieces were first cleaned using tetrahydrofuran, followed by bonding with the phosphate adhesive. The size of the specimen was 60 × 20 × 3 mm^3^ (Figure 1), which was prepared according to the ASTMD1002-10 standard [22]. Specimens were put into an oven and cured at the designated temperature. The performance of the SiC and phosphate adhesive is listed in Table 1.

Figure 1 shows the geometry of the specimens, with the characteristic dimensions defined as (in mm) $L_{o}$ = 10, lap width *b* = 20, total length between grips $L_{T}$ = 110, and adherents thickness $t_{P}$ = 3.

**Figure 2:**
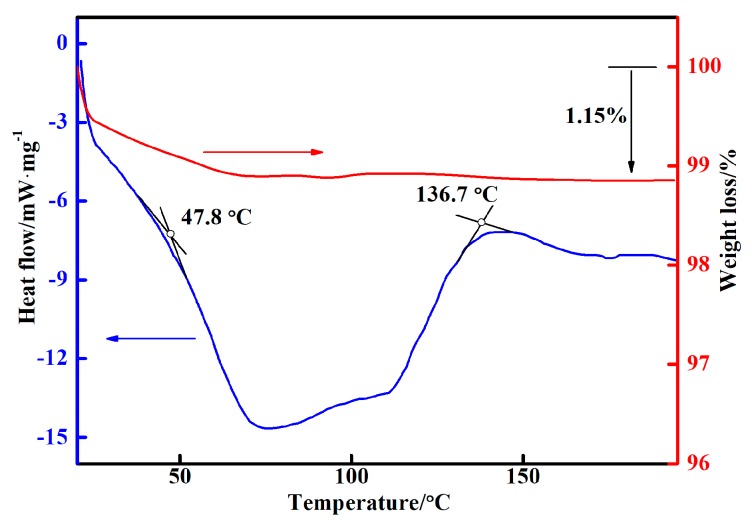
DSC and TG curve of the inorganic phosphate adhesive.

**Figure 3:**
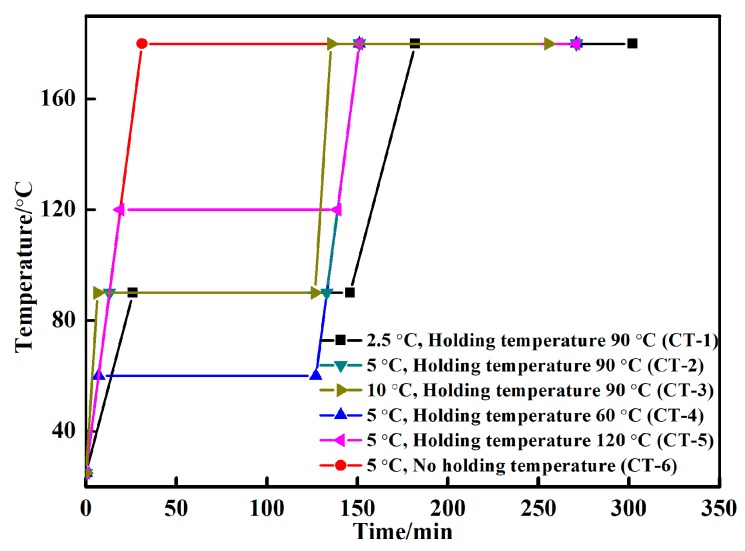
Curing temperature curves of the inorganic phosphate adhesive.

To design the curing temperature curves, differential scanning calorimetry (DSC) and thermogravimetric (TG) curves were used to test the inorganic phosphate adhesive (Figure 2), and the curing temperature interval of the phosphate adhesives was found to be between 47.8 °C and 136.7 °C. When the temperature increased, the TG curve decreased, which could be explained by the volatilization of water, resulting in weight loss. According to the curing temperature interval, we designed 6 curing temperature curves (Figure 3). When the heat preservation temperature was 90 °C, the heating rate was determined at 2.5 °C/min, 5 °C/min and 10 °C/min, respectively. When the heating rate was 5 °C/min, the heat preservation temperature was determined at 60 °C, 120 °C and without heat preservation for comparison.

### 2.2. Mechanical Characterization

**Figure 4:**
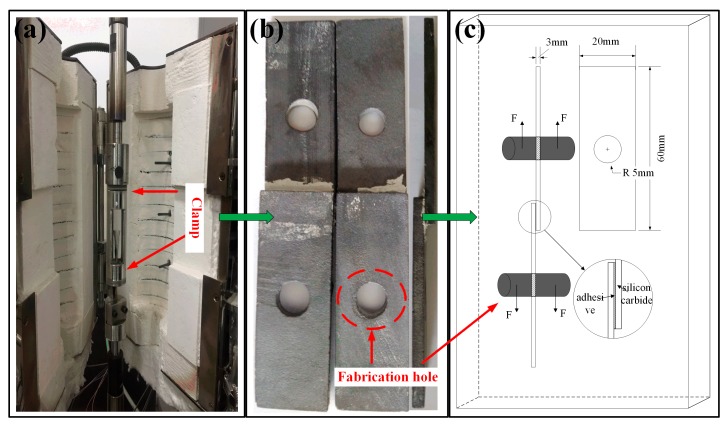
Shear strength test of the SLJ. (**a**) fixture diagram; (**b**) fabrication hole in specimens; (**c**) tensile test diagrammatic drawing.

The mechanical tests were performed at room temperature (RT) using an INSTRON 8032 tensile machine (INSTRON, Shanghai, China) as shown in Figure 4. Five specimens from each group were tested, and the average values were determined. In this experiment, the loading velocity of the stretching machine was 2 mm/min (ASTMD1002-10 [22]), and the loading process was halted once the tensile meter dropped to 20%. The shear strength of the adhesive is defined as

(1) $$T=\frac{P}{b\cdot L_{O}}$$

where *T* is the shear strength of adhesive and *P* is the maximum load for the sample shear failure.

### 2.3. Chemical and Structural Characterization

A scanning electron microscope (JEM1200EX, NEC Corporation, Tokyo, Japan) was used to observe the micro-topography. The adhesive was brittle fracture with liquid nitrogen, and the fracture surface was used as the test surface. A TG-DSC thermal analyzer (TG-DSC, STA449 F3, NETZSCH, Selb, Germany) was used to analyze the degree of reactivity effects at curing temperature. During the test, the heating rate was 10 °C/min in an argon environment with a mass flow of 30 mL/min from RT to 200 °C.

Micro-Raman spectroscopy (micro-Raman Microscope, Renishaw inVia, Gloucestershire, UK) was used to test the residual stresses. Micro-Raman spectroscopy consists of a Si laser used for exciting the specimen, a single spectrograph fitted with holographic notch filters, and an optical microscope (Renishaw inVia, Gloucestershire, UK) (Leica microscope with a motorized XYZ mapping stage) rigidly mounted and optically coupled to the spectrograph. The spectrometer (Renishaw inVia, Gloucestershire, UK) was first calibrated with a Si standard using a Si band position of 520.3 cm^−1^. A 100× objective lens was used to focus the incident beam (spot size ~1.5 μm) on the desired SiC grains and to collect the scattered beam from the specimen. Raman measurements were performed at room temperature, and the detailed parameters of the equipment are listed in Table 2.

**Figure 5:**
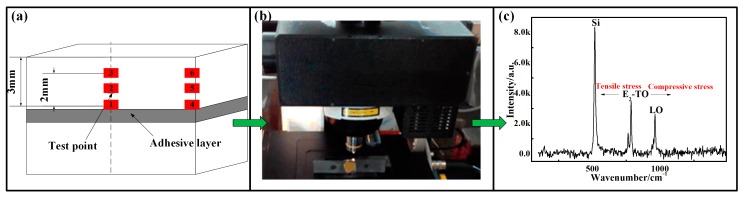
Schematic diagram of the residual stress test using micro-Raman spectroscopy. (**a**) test points; (**b**) testing process; (**c**) Raman spectrogram.

A schematic diagram of the residual stress test using micro-Raman spectroscopy is shown in Figure 5. Raman light reflects information pertaining to the elemental composition, lattice mass, and molecular structure of the material. Stress measurement with micro-Raman spectroscopy is based on the microscopic relationship between the Raman frequency shift and changes in atomic spacing as well as on the relation between the relative frequency shift and stress of specimens, which was expressed as follows:

(2) $$\sigma =\frac{2w_{o1}}{\left\{S_{12}(p+q)+S_{11}q\right\}}\Delta w_{1}=-K\Delta w_{TO}(MPa)$$

where $\Delta w_{TO}$ is the Raman frequency shift of the E_2_-TO peak [23].

## 3. Numerical Analysis

**Figure 6:**
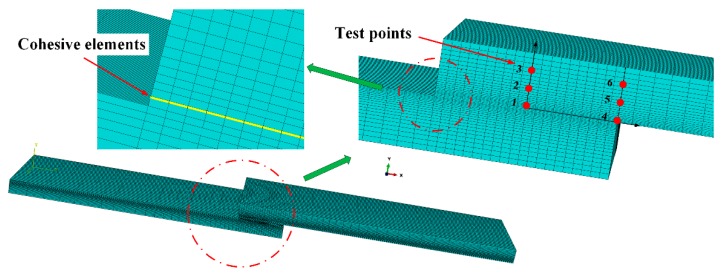
Mesh detail, test points and cohesive elements at the overlap for the $L_{o}$ = 10 mm adhesively bonded layer.

The FEM analysis was performed with an Abaqus^®^ during processing. Figure 6 shows the numerical model and mesh for the joint using Abaqus^®^; the points marked in red are the micro-Raman spectroscopy test points. Figure 6 also shows the mesh detail for the $L_{o}$ = 10 mm bonded joints at the overlap. The models used 8-node hexahedral solid elements (C3D8R from Abaqus^®^) and COH3D8 8-node cohesive elements. The joints were clamped at one end, while another end was subjected to tensile displacement with transverse restraining to simulate a tensile process. Part of the adhesive layer was modeled with the triangular CZM laws, incorporating a mixed-mode traction-separation law between the faces of the elements, including the stiffness of the adhesive layer.

**Figure 7:**
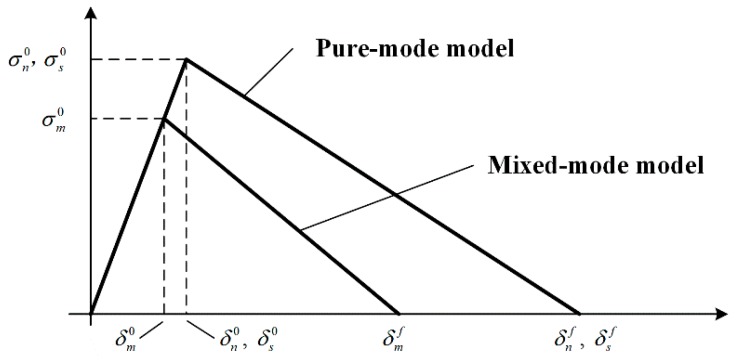
The traction–separation law with a linear softening law, available in Abaqus^®^.

The cohesive zone model is based on the relationship between stresses and relative displacements (in tension, shear or tearing) that connect paired nodes of the cohesive elements to simulate the elastic behavior up to the cohesive strength ($t_{n}^{0}$ in tension, $t_{s1}^{0}$ in shear or $t_{s2}^{0}$ in tearing) and subsequent softening to model the degradation of material properties up to failure. The triangular law (Figure 7) assumes an initial linear elastic behavior followed by linear degradation. Elasticity is defined by a constitutive matrix (*K*) containing the stiffness parameters and related stresses and strains across the interface:

(3) $$t=\left\{\begin{array}{l} t_{n}\\ t_{s1}\\ t_{s2} \end{array}\right\}=\left[\begin{array}{lll} k_{nn} & k_{ns1} & k_{ns2}\\ k_{ns1} & k_{s1s1} & k_{s1s2}\\ k_{ns2} & k_{s1s2} & k_{s2s2} \end{array}\right]\left\{\begin{array}{l} \varepsilon_{n}\\ \varepsilon_{s1}\\ \varepsilon_{2} \end{array}\right\}=K\varepsilon$$

A suitable approximation for the thin adhesive layers and debonding is provided by $K_{nn}=E$, $K_{s1s1}=K_{s2s2}=G$, $K_{ns1}=K_{ns2}=K_{s1s2}=0$, where *G* is the shear modulus, and the damage initiation can be specified by different criteria. Cohesive parameters of the adhesive for the cohesive damage model are listed in Table 3.

## 4. Results and Discussion

### 4.1. Residual Stress Analysis

**Figure 8:**
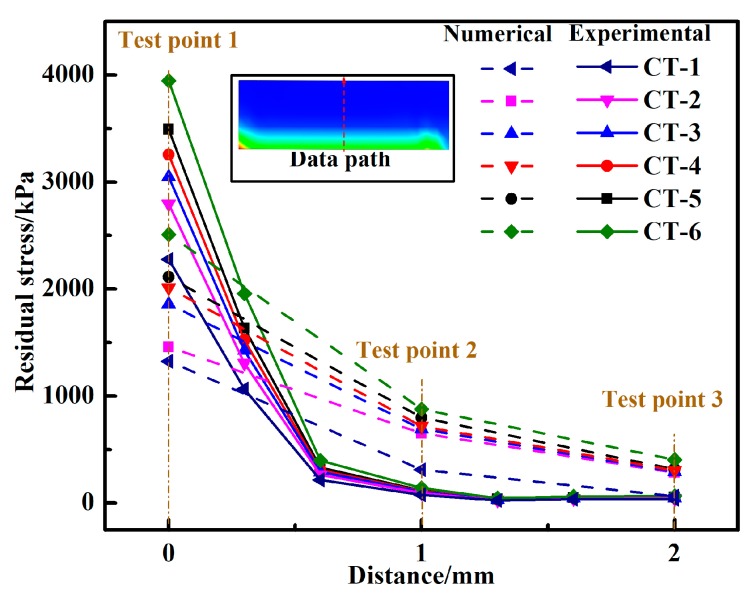
Test points’ experimental and numerical residual stress of the SLJ with different curing temperature curves.

Figure 8 shows the experimental and numerical residual stress of the 1-2-3 test points with different curing temperature curves, in which the numerical results agree with the experimental work. The residual stress decreases when the distance between the test point and adhesive layer increases. In addition, the simulated results are larger than the experimental results, such that we speculate that the error is due to the residual stress in the specimen, which was reduced by pre-test polishing and washing. When the curing temperature curve CT-1 was used, the residual stress was lower than that of the other specimens. When CT-6 was used, the residual stress of the specimen was at its highest. This phenomenon can be explained by the high heating rate, which accelerates the curing process and improves the crosslinking density. Due to the temperature difference between the lap end and center of the adhesive layer, the adhesive layer is partially cured. The viscosity of the adhesive is changed rapidly, which also lead to reduced fluidity. The stress caused by the chemical contraction of the adhesive layer is difficult to release, which results in an increase in residual stress. The residual stress of CT-4 is less than that of CT-5, which resulted from the stage of heat preservation caused by the pre-cured effect. In the early stage of the adhesive curing process, the molecules did not participate in the interface bonding, with the adhesive still in a viscous flow state. The stress in the adhesive layer is released and the residual stress is reduced. Although the CT-5 was heated for 2 h at the end of the curing process, the residual stress could not be released due to the high heating rate during the curing period.

### 4.2. Shear Strength Analysis

Table 4 lists the comparison of shear strength with the results in reference [24]. The failure modes of the specimens are cohesive failure. CT-1 has the highest shear strength (7.47 MPa) and CT-6 has the lowest shear strength (6.05 MPa). The shear strength of CT-2 was 7.25 MPa, which was close to those of CT-3 (7.08 MPa) and CT-1, and the shear strength of CT-4 (6.86 MPa) was higher than that of CT-5 (6.4 MPa). The results show that the curing temperature significantly influences the bonding strength of the phosphate adhesive. The heating rate and the heat preservation temperature also affected the bonding strength. When the heat preservation temperature was determined at 90 °C and the heating rate was varied, the highest shear strength of the specimen was CT-1, which was 5.51% higher than that of CT-3. When the heating rate was determined at 5 °C/min and the heat preservation temperature was different, the highest shear strength of specimen was CT-2, which was 19.83% higher than that of CT-6. The shear strength of RT-curing was higher than that of CT-5 and CT-6, which shows that the unreasonable curing temperature curves reduce the bonding strength.

**Figure 9:**
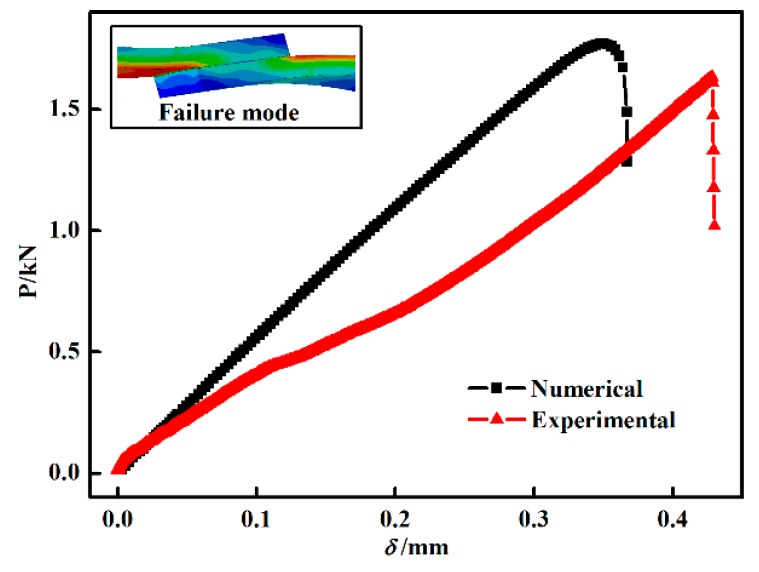
Comparison of P-δ curves for curing temperature curve (CT)-1 between the simulation and experiment.

Figure 9 presents a comparison of P-δ curves for CT-1 between the simulation and experiment in which the P-δ curves of the numerical and experimental results were in agreement. However, there is an obvious fluctuation during the starting period of the experimental curve. We speculate that this fluctuation is due to a slight slip of the SLJ end in the fixture as the tensile load increased.

### 4.3. Bonding Strength Prediction of SLJ

**Figure 10:**
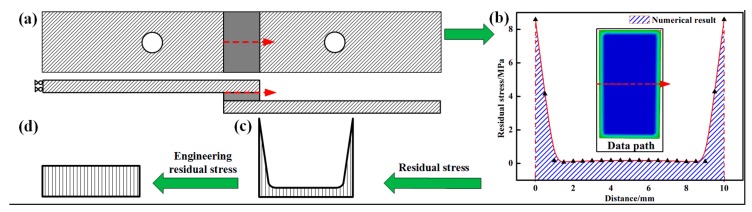
Schematic diagram of the residual stress transformation process to engineering residual stress. (**a**) specimen diagrammatic drawing; (**b**) residual stress distribution in test path; (**c**) residual stress; (**d**) engineering residual stress.

The residual stress distribution of the SLJ was simulated as shown in Figure 10b. The residual stress of the SLJ is approximately the distribution of the saddle function, and the residual stress is defined as follows in the application:

(4) $$\sigma_{e}=\frac{-K\Delta w_{TO}(x)|{}_{0}^{l}}{l}$$

where l is the length of the lap zone and $\Delta w_{TO}$ is the TO peak offset in Equation (2).

To predict the bonding strength of the SLJ, we defined the concept of engineering residual stress. Figure 10 shows the schematic diagram of the residual stress transformation process.

**Figure 11:**
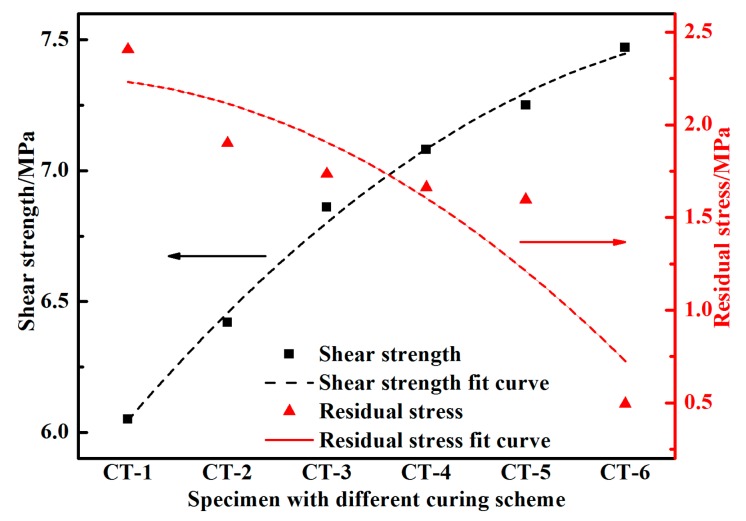
The residual stress effect of the SLJ on the shear strength.

The relationship between the residual stress and shear strength is shown in Figure 11. The residual stress in the adhesive bonded SLJ was correlated with the shear strength: The shear strength of the SLJ decreases with the increasing residual stress in the joint. In addition, the free end contraction at the edge of the adhesive layer leads to stress concentration near the lap end, reducing the adhesive bonding strength.

### 4.4. Microstructure Analysis

**Figure 12:**
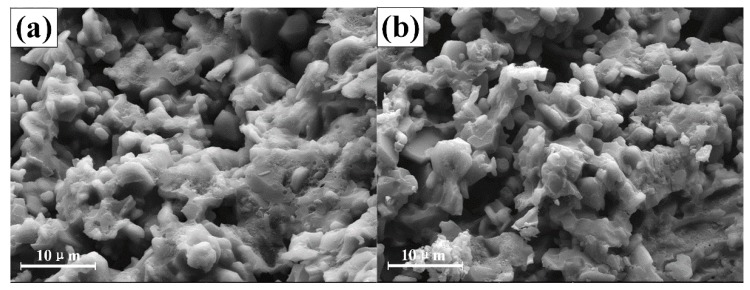
The microstructure of adhesive curing with the CT-1 (**a**) and CT-6 (**b**) temperature curve.

To further study the effect of the curing temperature curves’ mechanism on adhesive performance, the microstructure of the adhesive curing with different temperature curves (CT-1 and CT-6) was observed (Figure 12). As shown in the figure, there are many holes and fractures inside the adhesive. The microstructure of the adhesive in Figure 12a is relatively flat, and the pores and cracks are evenly distributed in the adhesive layer. Furthermore, we did not observe an obvious difference between the CT-1 and CT-6 specimen. Therefore, the two curing temperature curves had little influence on the microstructure of the phosphate adhesive, in which the primary reason for the decrease of shear strength is still the increase in residual stress.

## 5. Conclusions

In this paper, we aimed to determine how the curing temperature curves affect the tensile shear strength of an SLJ bonded with phosphate adhesive using six designed curing temperature curves. The performance of these specimens was tested and simulated, and the following conclusions were drawn:

The curing temperature affects the bonding strength and has little effect on the microstructure of the phosphate adhesive; the curing temperature directly affects the residual stress forming process of the SLJ; the residual stress affects the bonding strength of the phosphate adhesive; and the designed curing temperature curves increase the bonding strength by up to 23%. These conclusions can provide references for the future application of the phosphate adhesive.

## Figures and Tables

**Table 1:** Performance of the SiC and phosphate adhesive.

Parameter	Adhesive	Silicon Carbide (SiC)
Thermal conductivity (W/m °C)	≤0.52	3.2
Density (g/cm^3^)	2.21	3.22
Specific heat (J/kg °C)	585.152	399.84
Coefficient of thermal expansion (/°C)	1.73 × 10^−6^	4.7 × 10^−6^
Elongation at break (%)	2.2	
Modulus of elasticity (GPa)	48.12	330

**Table 2:** Micro-Raman spectroscopy parameters.

Laser Wavelength (nm)	Laser Power (mW)	Exposure Time (s)	Scan Time (s)	Step Size	Estimated Dimensions (um)
532	5.0	0.01667	20	10	20

**Table 3:** Cohesive parameters of the adhesive for the cohesive damage model.

Property	Adhesive
*E* (GPa)	48.12
*G* (GPa)	18.51
$t_{n}^{0}$ (MPa)	17.63
$t_{s1}^{0}=t_{s2}^{0}$ (MPa)	14.9
$G_{n}^{c}$ (N/mm)	0.43
$G_{s1}^{c}=G_{s2}^{c}$ (N/mm)	4.70

**Table 4:** Comparison of shear strength with results in reference [24].

Specimen	Shear Strength (MPa)	Error (MPa)	Increment Rate (%)
CT-1	7.47	±0.51	+23.47
CT-2	7.25	±0.22	+19.83
CT-3	7.08	±0.46	+17.02
CT-4	6.86	±0.52	+13.39
CT-5	6.42	±0.37	+6.12
CT-6	6.05	±0.55	0
RT-curing	6.79	±0.30	/
